# The Effect of Mixture Proportion on the Performance of Alkali-Activated Slag Concrete Subjected to Sulfuric Acid Attack

**DOI:** 10.3390/ma15196754

**Published:** 2022-09-29

**Authors:** Mohammad Teymouri, Kiachehr Behfarnia, Amirhosein Shabani, Armin Saadatian

**Affiliations:** 1 Department of Civil and Environmental Engineering, Colorado State University, Fort Collins, CO 80523, USA; 2 Department of Civil Engineering, Isfahan University of Technology, Isfahan 84156-83111, Iran; 3 Department of Civil Engineering and Energy Technology, Oslo Metropolitan University, Pilestredet 35, 0166 Oslo, Norway; 4 Department of Construction Management, Colorado State University, Fort Collins, CO 80523, USA

**Keywords:** alkali activated slag concrete, sulfuric acid, mix design parameters, AASC, durability

## Abstract

Long-term deterioration and durability concerns in harsh environments with acidic attacks are considered as the weaknesses of ordinary Portland cement (OPC) concrete. Although the performance of alkali-activated slag concrete (AASC) has been reported to be superior in acidic environments, there is a poor understanding regarding the impacts of diverse mix design parameters on AASC durability in an acidic environment. This research aims to understand the impact of mix design parameters on the durability of AASC in the sulfuric acid (H_2_SO_4_) environment with pH = 3. The type of alkaline solution, the molarity of alkaline solutions, the weight ratio of alkaline solutions to slag, and the weight ratio of NaOH to Na_2_SiO_3_ are mix design parameters investigated in this study. The compressive strength reduction and weight loss were monitored from early ages up to 180 days. Moreover, an OPC concrete sample was produced as a reference.

## 1. Introduction

High energy consumption and carbon dioxide emission during production (around 5% to 7% of all CO_2_ emissions) are the main disadvantages of ordinary Portland cement (OPC) concrete [1]. Long-term deterioration and durability concerns in harsh environments such as acidic attacks are other drawbacks of OPC concrete [2,3]. In the sewer system, the sulfates in the wastewater convert to H_2_S. Absorption of H_2_S onto the moist upper surfaces of the concrete pipe results in the formation of one of the most prevalent acids in the environment, H_2_SO_4_, and other sulfur byproducts [4]. In acidic environments, OPC concrete is penetrated by hydronium ions (H_3_O), resulting in a significant drop in pH levels of pore solutions. A drop in the pH level of pore solution in OPC concrete results in the decomposition of calcium hydroxide (CH), calcium silicate hydrate (CSH), and ettringite at the pH of 12.6, 10.7, and 10.5, respectively [5]. Continuation of this process results in the dissolution and/or decomposition of components such as CH and CSH and influences the chloride binding mechanism [6]. A product of such reactions, calcium salts, will reduce the compressive and flexural strength of OPC concrete. Alkali-activated concrete (AAC) has been considered a potential replacement for OPC concrete and one of the methods to produce more durable and environmentally friendly concrete [7,8,9,10,11,12]. This is due to the high chemical resistance of their alternative binders compared to traditional cementitious binders [13,14]. 

AAC is a cement-free concrete containing alumina-silica sources, such as fly ash and slag, and alkaline solutions [14,15]. Alkaline activation of different industrial byproducts such as slag, fly ash, and natural minerals produce these alternative binders that possess cementitious properties [15]. Activation of slag with an alkaline solution such as sodium silicate (Na_2_SiO_3_) and sodium hydroxide (NaOH) can produce alkali-activated slag concrete (AASC) that has been reported to have superior acid resistance performance [16,17]. Properly designed AASC mortars showed sulfuric acid resistance, but the addition of GGBFS led to the formation of expansive calcium sulfate resulting in a decrease in acid resistance of AASC samples [18]. Slag is a byproduct of the steel making industry [19] that is used as a supplementary cementitious material (SCM) in conventional concrete and as an alumina-silica source in AASC. AASC can also be produced using similar equipment to those available for OPC concrete production. AASC does not have CH in its hydration products that react with acid agents and form expansive acid salts [3,5]. In addition, the highly cross-linked three-dimensional aluminosilicate structure of AASC provides good performance for these binders in acidic solutions [20].

A wide range of mix design parameters affects the acid resistance of AASC. The type of ion in alkali activator [21], the application of nano-silica or micro silica modifications that improve the microstructure of the matrix [22], and the chemical composition of aluminum silica materials such as slag, fly ash, and micro-silica [4,17,23,24] are some of the underlying factors that can influence the acid resistance of AASC. The most effective activator used in development of high-strength AASC is Na_2_SiO_3_ solution [16,25,26]. Previous research efforts confirmed that using Na_2_SiO_3_ with proper Na_2_O content and silica modulus creates high-strength AASC [16,25,26]. The predominance of CSH and the volume of pores in the size of the mesopores range characterize the blast furnace slag concrete activated with sodium silicate. Hence, the development of high-strength concrete using Na_2_SiO_3_ and NaOH is recommended [27,28]. AASC is reported to achieve a compressive strength of more than 80 MPa at 28 days [16,26,29]. Although the application of alkaline solutions contributes to CO_2_ emissions [30,31,32], the AASC emission level could be significantly lower than OPC concrete if formulated properly [33]. 

In the mix design of AASC, molarity and type of alkaline solutions are two essential elements affecting the mechanical properties and durability of the product [34]. In other words, variance in the chemical composition of slag may affect its durability, which may have a direct impact on concrete resistance in acidic environments [35]. The impact of mix design parameters on the mechanical properties and durability of AASC in acid attack for up to 56 days due to sulfates, chlorides, and nitrates was investigated in [36]. Their results indicated that NaOH molarity has the highest level of impact on the mechanical strength and durability of AASC [36]. Influential factors on the workability and mechanical properties of fly ash and slag-based geopolymer concrete were studied in [37]. It was concluded that an increase in the molarity of NaOH solution and slag content coupled with a decrease in alkaline activator solution improved the compressive strength of the concrete [37,38]. AASC is more resistant to acid deterioration compared to OPC concrete of a similar grade, and the predominant deterioration mechanism was CSH declassification and the formation of a soluble salt (calcium acetate) [18]. The sulfuric acid resistance of alkali-activated mortars using different silica materials, including micro silica and rice husk ash, was investigated in [23]. The ground granulated blast furnace slag (GGBFS) was to mix design samples as a source of calcium. The effect of mix design on the performance of AASC in HCl acid attack is evaluated in [29]. Although researchers investigated the diverse properties of AASC [39,40], there is a poor understanding regarding the impacts of diverse mix design parameters on AASC durability in an acid environment.

The ever-increasing demand for more durable concrete compared to OPC concrete validates the research for the performance of alternative binders in such a harsh environment. Acid-resistant concrete is needed in acidic environments such as sewer and agricultural structures, biogas plants, and marine structures. In the review of the existing literature, a limited number of studies focusing on a systematic approach to understanding the effect of AASC mix design parameters on its performance and durability in harsh acidic environments were identified. Moreover, the impacts of acid attacks on the short-term and long-term performance of AASC have been investigated. Nevertheless, there is a poor understanding regarding the impacts of diverse mix design parameters on AASC durability in the sulfuric acid environment. This research aims to understand the impact of four main mix design parameters: molarity of alkaline solutions, type of alkaline solution, the weight ratio of alkaline solutions to slag, and the weight ratio of alkaline solutions to sodium silicate, on the durability of AASC samples. The samples were submerged in H_2_SO_4_ of pH = 3 for up to six months, and variations in compressive strength and weight loss were recorded at 7, 14, 28, 90, 120, and 180 days. The results show that an increase in the alkaline solution to slag ratio increased compressive strength reduction and weight loss. In addition, the application of KOH-activated slag concrete negatively affected mechanical performance and increased the production cost of AASC.

## 2. Materials and Test Procedure

### 2.1. Material Properties

In this study, GGBFS with a specific gravity of 2.85 gr/cm^3^, Blaine fineness of 400 m^2^/kg, the Al_2_O_3_/SiO_2_ weight ratio of 0.447, and CaO/SiO_2_ weight ratio of 1.079 was used as a silica-aluminate source. OPC concrete specimens were produced as reference concrete. Table 1 shows the oxide content of GGBFS and OPC. Hydration modulus (HM = (CaO + MgO + Al_2_O_3_)/SiO_2_) is an important parameter regarding the reactivity of GGBFS. It was reported that HM should be greater than 1.40 to guarantee a satisfactory hydration property [41]. In this study, HM was 1.77 based on oxide contents for GGBFS. 

A combination of NaOH (as the most commonly used activators) and Na_2_SiO_3_ or KOH and Na_2_SiO_3_ were used as alkaline solutions. To evaluate durability and the performance of AASC after acid attacks, both NaOH- and KOH-activated slag concrete were cast and monitored in this study. NaOH and KOH with a purity of 98% are white flake solids. They were dissolved in water to make a solution with a desirable concentration at least 15 min prior to the commencement of the mixing process. Three concentrations of 6, 10, and 14 were used for NaOH and KOH solutions. Na_2_SiO_3_ was in liquid form with a SiO_2_/Na_2_O ratio of 2.5, water mass content of 51%, and Na_2_O and SiO_2_ contents were 14% and 35%, respectively. Limestone aggregates are widely used in most projects in Iran due to their lower cost and abundance. Thus, coarse aggregate (crushed limestone) with a maximum aggregate size of 19.5 mm and fine aggregate (crushed sand) were used in this study. The aggregate grading was as per ASTM C33. Table 2 presents the physical properties of aggregates. Sand equality, water absorption of fine aggregates, specific gravity in saturated surface dry (SSD) condition, and water absorption of coarse aggregates were measured according to ASTM D2419, ASTM C128, and ASTM C127, respectively. 

### 2.2. Mix Proportions and Casting

The mix design procedure of AASC was selected based on [42]. In all mix designs, the water-to-solid materials ratio and weight percentage of the aggregates were 0.50 and 77%, respectively, considering the water content of NaOH solution when mix proportions were calculated. To have a constant workability for all AASC specimens, a naphthalene-based superplasticizer (SP) was used. In this study, the four selected parameters were the type of alkaline solution, the molarity of alkaline solutions, the weight ratio of alkaline solutions to slag (A/S), and the weight ratio of NaOH (or KOH) to Na_2_SiO_3_ (NO/NS). Based on these parameters, 15 mix designs and 540 cubic specimens (100mm × 100mm × 100 mm) were made. Table 3 shows mix codes and corresponding proportions.

NaOH and KOH solutions were prepared at desirable molarity prior to the mixing process and were added to the mixture in liquid form. It should be noted that alkaline solutions were made 30 min prior to addition to the mixture. Therefore, their temperature almost reached the ambient temperature. A sixty liters mixer was used for mixing the materials. The mixing process was as follows: First, aggregates and GGBFS were mixed for 3 min. Then, the alkaline solutions were gradually added to the blended materials. Finally, water and SP were added to the mixture. Subsequently, the materials were mixed for 5 min, and then, the mixture was allowed to rest for 1 min. Finally, the mixture was remixed for another 3 min. 

The prepared mixture was poured into cubic samples, and to minimize water evaporation, the molds were covered with plastic sheets in the first 24 h at a controlled temperature of 23 ± 2 Celsius. Reference OPC concrete samples were also made. OPC concrete samples were made using the same aggregates used in AASC mixtures. The water-to-cement ratio was set at 0.28, and the same SP was used to enhance the workability of specimens. OPC concrete samples were cured in water for 28 days before exposure to sulfuric acid and were designed to have comparable compressive strength (60 MPa at 28 days) to AASC specimens. Table 3 presents mixed design proportions of AASC samples.

### 2.3. Curing Methods and Test Procedure

The molds were placed in a water bath for two weeks before transferring them to an H_2_SO_4_ bath. It should be noted that this curing time (two weeks) is enough for AASC samples to gain nearly 80% of their 180 days of compressive, as will be discussed in Section 3.1. After two weeks of water curing, the specimens were removed from the water and kept in a room with a controlled temperature of 23 ± 2 Celsius for three hours. Prior to a weight measurement, specimens were sandpapered on all six sides. The samples were weighed before immersing in an H_2_SO_4_ bath with a pH of 3. To keep the pH level constant, the pH of the bath was measured with strips pH meter twice a week. In case of disparity in pH level of the bath, H_2_SO_4_ 98% was added.

As stated earlier, a standard procedure for assessing acid attacks on concrete is yet to emerge. In this study, the acid resistance of AASC and OPC concrete was tested by exposing the samples to an H_2_SO_4_ solution with a pH of 3 for up to six months. The pH was selected based on [18,21,35,43]. Subsequently, reduction in compressive strength and weight loss were measured to address the study’s main objective. At the age of 28, 90, 120, and 180 days, three replicate cubes from each mix design were removed from the acid and water baths for the compressive strength test and weight loss measurement. The reduction in compressive strength (RCS) was calculated using the following equation:

$$\mathrm{RCS}=\frac{A-B}{A}$$

where A is the average compressive strength of three cubic samples cured in potable water, and B is the average compressive strength of three cubic companion samples cured in an H_2_SO_4_ bath. The compressive strength test was conducted as per EN 12390-3 at predetermined intervals. Furthermore, the chemical compositions of slag and Portland cement were measured using the X-ray fluorescence (XRF) test.

## 3. Results and Discussion

### 3.1. Mix Design Effects on Compressive Strength of AASC

**Figure 1:**
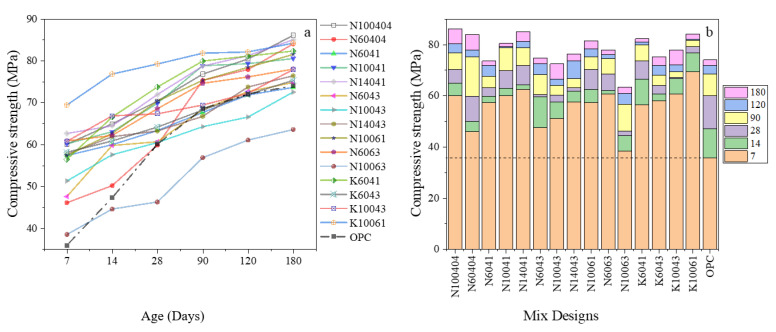
(**a**) Compressive strength development; (**b**) Compressive strength obtained at different ages of OPC and AASC samples before acid exposure.

Figure 1a shows the compressive strength development of AASC and OPC mixes cured in water for up to 180 days. As shown in this graph, changing the mix design parameters drastically impacts strength development from an early age. The OPC specimens, as expected, showed a sharp strength development up to 90 days, from nearly 36 MPa at 7 days to 68.5 MPa at 90 days. After that, the compressive strength of OPC only increased by about 8%. KOH-activated slag concrete showed higher early and final compressive strength compared to OPC and NaOH-activated slag samples. 

Figure 1b depicts how much compressive strength was obtained at varying ages. After 28 days of curing in water, the compressive strength for AASC samples ranged from 60 MPa to 79.2 MPa. However, the N10063 mixture showed lower strength from 14 days compared to OPC concrete. Low strength of mix code N10063 is associated with simultaneous adverse effects of a high A/S ratio (0.6) and No/NS ratio (3), leading to a considerable amount of water in the mix design and reducing compressive strength. 

The general trend was that AASC gained considerably high strength in the early ages. If we consider the compressive strength at 180 days as the ultimate strength, samples containing KOH gained more than 80% of their final strength after two weeks. Higher molarity and A/S ratio of KOH-activated slag specimens resulted in the highest compressive strength. 

Apart from N60404, the inclusion of NaOH also resulted in archiving at least 75% of the ultimate strength at 14 days. The results clearly indicated that selecting a proper mix design proportion for AASC would lead to having substantially high compressive strength values. The underlying reason is attributed to reaction kinetics and microstructure development in AASC. Compared to Portland cement, slag contains higher silica (SiO_2_), almost 38%, two times more aluminum oxide (Al_2_O_3_), and roughly 40% lower calcium oxide (CaO) contents. Unlike Portland cement, CH is not a product in the AASC structure [14]. Slag is amorphous, and its hydration leads to the formation of calcium silicate hydrates (CSH) which have high silica content. In addition, three other hydration products are hydrotalcite, ettringite, and monosulfate [44]. 

Highly alkaline environments provided by alkaline solutions resulted in the very fast dissolution of the slag of AASC and formed hydration products in the early hours. The rapid formation of hydration products for AASC was reported after 3 h [44]. The type of activator and its molarity directly impact the rate of hydration products and microstructure development. 

**Figure 2:**
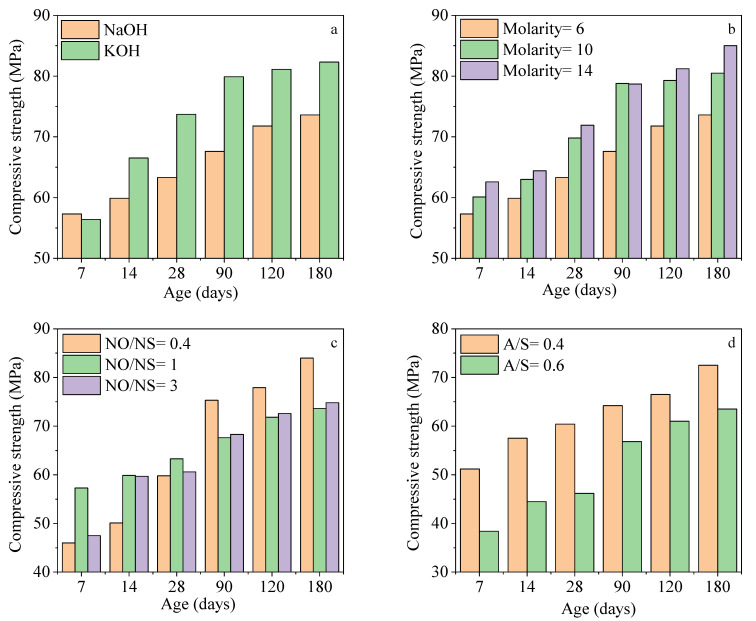
The effects of mix design parameters on compressive strength: (**a**) solution type (molarity = 6, A/S = 0.4, NO/NS = 1), (**b**) molarity (Solution = NaOH, A/S = 0.4, NO/NS = 1), (**c**) NO/NS ratio (Solution = NaOH, molarity = 6, A/S = 0.4), and (**d**) A/S ratio (Solution = NaOH, molarity = 10, NO/NS = 3).

Figure 2 depicts the impacts of four mix design parameters on strength development. As illustrated in Figure 2a, KOH activated slag achieved higher strength after 7 days of curing than the NaOH-activated slag concrete, and the differences between compressive strength at 28 and 90 days are more than 10 MPa. Thus, KOH is more promising compared to NaOH in terms of compressive strength results. The KOH solution, due to a higher molecular mass [45] and degree of hydration [46] compared to NaOH, increased the compressive strength. The results showed that when the A/S and NO/NS ratios were constant, an increase in molarity of NaOH from 6 to 10 and then to 14 improved the compressive strength constantly from early ages to 180 days (Figure 2b). The main reason is that a higher concentration of alkaline solutions increased the reaction rates and thus led to higher compressive strength values. Higher solution concentrations mean an increase in solid NaOH in the solution leading to an increase in the rate of ionization of slag particles and the rate of crystallization of products. Moreover, higher solution molarities would increase the solubility of anions (silicate and aluminate) and cations (calcium) in slag. 

In NaOH-activated slag samples with a molarity of 10 and A/S ratio of 0.4, the higher ratio of NO/NS reduced the strength after 90 days, as illustrated in Figure 2c. The same trend was observed at a molarity of 10 and an A/S ratio of 0.4 after 28 days. The main reason is that NaOH contains a high volume of water which negatively impacts the microstructure, and the excessive water forms higher porosity in the AASC microstructure leading to lower compressive strength. Moreover, Na_2_SiO_3_ is a rich source of silica which can contribute to forming higher content of CSH in the concrete microstructure. Hence, a low NO/NS ratio (higher Na_2_SiO_3_) in solution would potentially increase the CSH in the concrete leading to higher mechanical properties. Roughly 77% of NaOH and 52% of Na_2_SiO_3_ consist of water. The lowest NO/NS ratio (0.4) contains higher Na_2_SiO_3_ content due to its high viscosity and reduced concrete workability. Slag needs enough alkaline solution to react, and the excessive amount of alkaline, which consists of a considerable amount of water, causes capillary cavities in the microstructure and negatively impacts the compressive strength. Figure 2d displays that the lower the A/S ratio, the higher the compressive strength. The difference in compressive strength was even higher at early ages. The main reason is that when the A/S ratio increases from 0.4 to 0.6, the amount of binder is reduced. Thus, there is not enough binder to cover aggregates and form an integrated structure which was also reported in [16,47,48].

OPC concrete needs sufficient water curing and time to progress its hydration products and form the concrete structure. However, AASC, given an alkaline solution for alumina silicate powder, substantially increases the rate and intensity of reactions leading to obtaining high mechanical properties in the early ages. The water absorption was also measured for AASC samples which were between 2.3% to 3.88%. The KOH-containing specimens showed higher water absorptions compared to NaOH-containing samples. The water absorption for KOH-activated slag concrete was more than 3%, and NaOH-activated slag had an average water absorption of 2.7%. 

### 3.2. Mix Design Effects on the Performance of AASC in the Acidic Environment

#### 3.2.1. Type of Alkaline Activator

**Figure 3:**
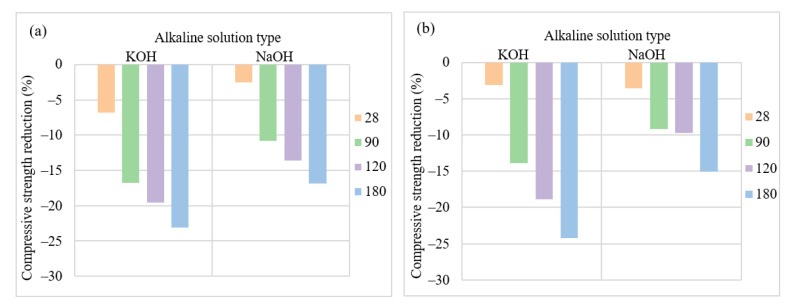
The effects of KOH replacement on compressive strength reduction at a molarity of 6, A/S = 0.4 and (**a**) NO/NS = 1, and (**b**) NO/NS = 3.

Based on the literature review, the most commonly used activators are NaOH and Na_2_SiO_3_ [16,18,31,49] due to their reasonable cost and promising results. In this paper, all mix designs contained a combination of two alkaline solutions, NaOH and Na_2_SiO_3_ or KOH and Na_2_SiO_3_. Brough et al. [45], Ye et al. [50], and Park et al. [51] reported KOH-activated slag as an alternative option for NaOH-activated slag concrete with comparable mechanical and durability results. The application of KOH in AASC faces a severe challenge mainly due to its cost. Due to the high cost of activators, OPC concrete is preferred in construction [52]. Thus, selecting a proper mix design for AASC, including the type of activator and its concentration, can play a key role in cost analysis [53]. The corrosive alkaline solutions can cause severe challenges in transportation, storage, and mixing with slag. Moreover, in terms of durability in acidic attacks, KOH inclusion in the AASC mix design did not provide promising results based on Figure 3.

Figure 3 displays the compressive strength reduction of AASC containing KOH and NaOH after acid exposure up to 180 days. The results revealed that KOH-activated slag samples experienced higher compressive strength reduction at all ages. Although AASC mixes containing KOH showed higher compressive strength after water curing in all ages than NaOH-activated slag concrete, their performance in sulfuric acid solution was inferior. When NO/NS = 1, KOH-activated slag showed a 6.24% higher strength reduction after 180 days of exposure to acid compared to NaOH-activated slag concrete, as illustrated in Figure 3a. When NO/NS = 3 (see Figure 3b) difference between the strength reduction of samples containing KOH and NaOH is 9.19% after 6 months of immersion in the acid solution. The core cause is that KOH reacts with slag particles and an intense exothermic reaction occurs that forms spherical hydration products, which are not uniformly dispersed in the structure [48].

Hence, countless unfilled voids and cavities are created in microstructures that are mainly connected. When acid enters such a porous structure, cavities are filled with acid agents, and the acid moves through connected porosity all around the concrete structure leading to reactions with hydration components and severe deterioration [17]. As a result, the incorporation of KOH creates a less homogeneous microstructure. The replacement of KOH at a molarity of 10 and NO/NS ratio of 3 was also tested. However, due to the resilience of the results, they are not mentioned in this section. 

**Figure 4:**
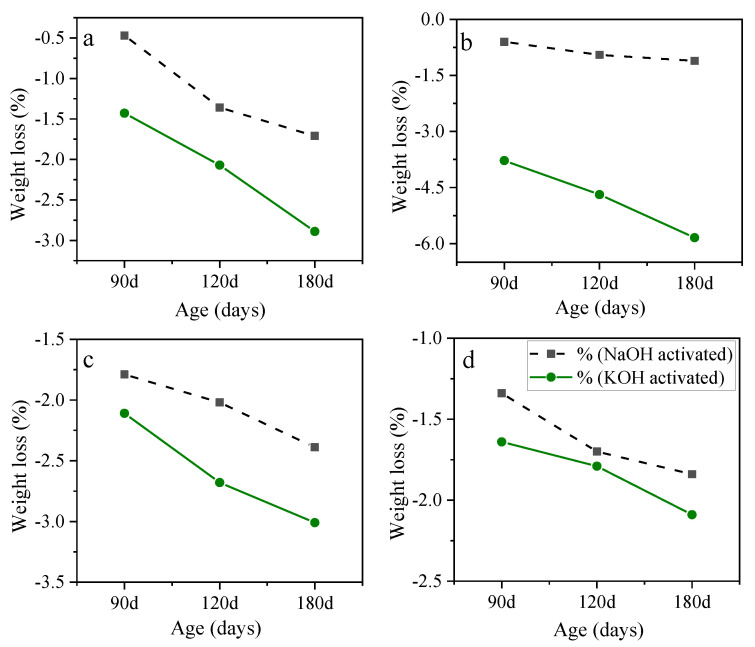
The weight loss of samples with (**a**) A/S = 0.4, molarity = 6 and NO/NS = 1, (**b**) A/S = 0.4, molarity = 6 and NO/NS = 3, (**c**) A/S = 0.6, molarity = 6, and NO/NS = 1, and (**d**) A/S = 0.4, molarity = 10 and NO/NS = 3.

Figure 4 shows the weight loss reduction for AASC samples containing KOH and NaOH at different molarities, A/S, and NO/NS ratios. As illustrated in Figure 4a, the replacement of KOH at a molarity of 6 and NO/NS of 1 caused higher weight loss. AASC samples containing KOH experienced roughly 47% and 34% higher weight loss than NaOH-activated slag samples at the age of 120 and 180 days, respectively. The results also showed that the replacement of KOH at a molarity of 6 and NO/NS of 3, Figure 4b, considerably increased weight loss which was 5.3 times greater than specimens made with NaOH. The comparison was also made using an A/S ratio of 0.6, Figure 4c, and a molarity of 10, Figure 4d, which resulted in the same trend. The worse results with KOH inclusion can be attributed to the pore microstructure of KOH-activated slag concrete, as discussed earlier. KOH application as an activator, due to the nature of its reaction with slag, caused a porous microstructure and made it more vulnerable to acid attacks. 

**Figure 5:**
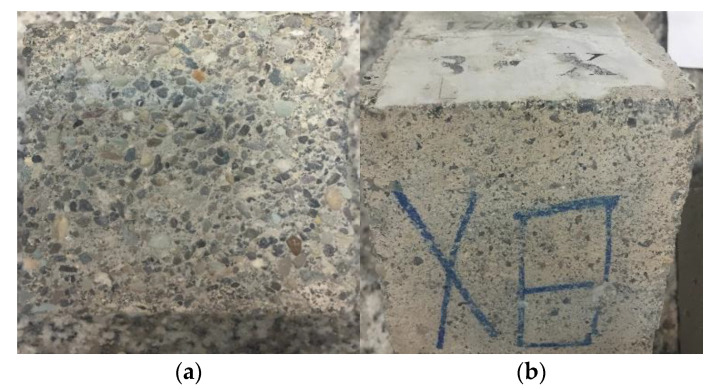
(**a**) KOH and (**b**) NaOH-activated slag concrete specimens exposed to H_2_SO_4_ after 6 months.

Visual inspection clearly shows that samples containing NaOH and Na_2_SiO_3_ performed better in the acidic environment than specimens containing KOH and Na_2_SiO_3_. Figure 5 shows KOH-activated slag concrete samples exposed to acid for 6 months. The surface layer leached out, and the main part of the binder was removed in all six faces. Moreover, as discussed in Section 3.1, samples activated by KOH have higher water absorption, meaning they have higher porosity and permeability. Hence, when the samples were exposed to acid, more acids could ingress the specimens, leading to higher deterioration.

#### 3.2.2. NaOH Molarity

**Figure 6:**
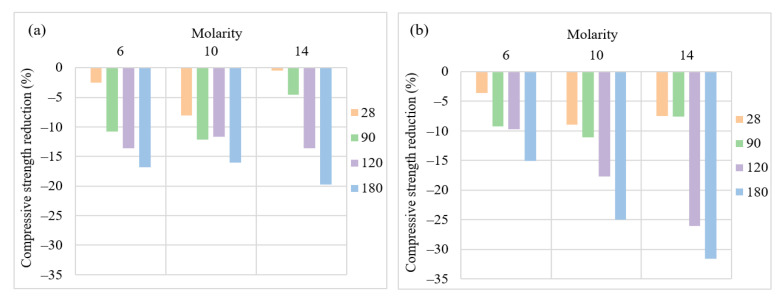
The effects of NO/NS ratio of compressive strength reduction after acid exposure (**a**) NO/NS = 1 and (**b**) NO/NS = 3.

Figure 6 shows the impacts of change in molarity of NaOH from 6 to 14 on the compressive strength of AASC after acid exposure for up to six months. The increase in NaOH concentration means an increase in solid NaOH in solution. Although the compressive strength of samples containing a higher molarity of NaOH cured in water increased, as discussed in Figure 2b, the trend was not the same when samples were exposed to the acid solution. The increase in molarity was twofold. First, it brought an increase in the crystallization rate of products in NaOH-activated slag. Moreover, more CSH forms and denser microstructures were created in the No/NS ratio of 1 compared to 3 due to the higher amount of Na_2_SiO_3_. Secondly, at higher molarity, the rate of reaction and the amount of ionization of slag particles uncontrollably increased, leading to the formation of an irregular structure in concrete. 

The effects of molarity change were investigated in two NO/NS ratios of 1 and 3. In the NO/NS ratio of 3, Figure 6b, due to the lower content of Na_2_SiO_3_ as a source of Si for the formation of CSH compared to the NO/NS ratio of 1, the impacts of irregular and weak microstructure were more significant than higher crystallization of slag particles. Another factor that should be taken into consideration is that the higher molarity means a higher production cost which is not favorable.

**Figure 7:**
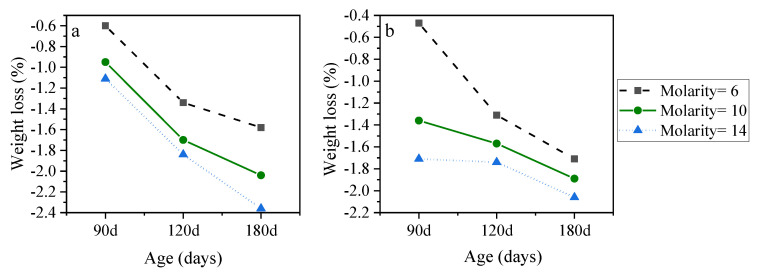
The effects of NaOH molarity on weight loss of AASC with (**a**) NO/NS = 1 and (**b**) NO/NS = 3 after acid exposure.

Figure 7 displays the weight loss of AASC samples when NaOH concentration changes from 6 to 14 at two NO/NS ratios of 1 and 3. It should be noted that after nearly 60 days of exposure to acid, a soft white layer was formed on the surface of the samples. This loose layer was removed from the samples before weighing; otherwise, the weight loss would have been lower after 180 days of acid exposure compared to 120 days owing to the increasing formation of this white layer. The results showed that the lowest weight loss was observed at all immersion ages at a molarity of 6. The trend was the same in two NO/NS ratios of 1 and 3. In both ratios, the molarity of 14 caused higher weight loss compared to lower concentrations. The main reason is that the formation of irregular structures due to more intensive reactions at high concentrations causes pore structure contents to easily leach out when exposed to acid agents. Therefore, based on the results, increasing NaOH concentration is not economically desirable and causes more compressive strength reduction and weight loss in the sulfuric acid environment, especially when NO/NS is higher than 1.

#### 3.2.3. NaOH to Na_2_SiO_3_ Ratio

**Figure 8:**
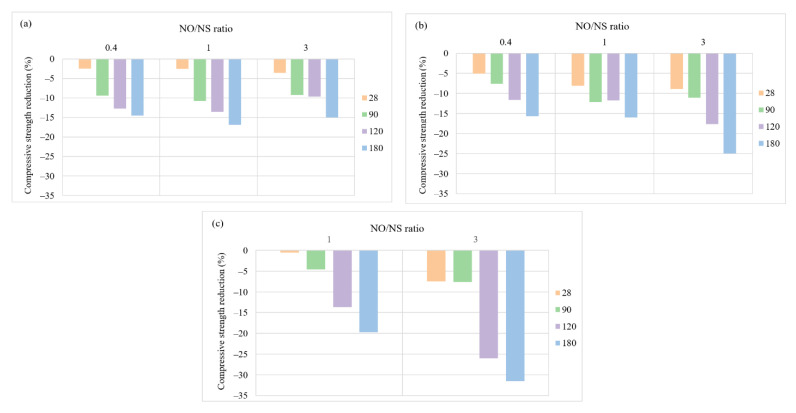
The effects of NO/NS ratios on compressive strength reduction at a molarity of (**a**) 6, (**b**) 10, and (**c**) 14.

The impacts of the NO/NS ratios ranging from 0.4 to 3 on the performance of AASC samples immersed in sulfuric acid solution with pH = 3 were investigated at A/S = 0.4 and the three different NaOH molarities of 6, 10, and 14, as shown in Figure 8. The effects of increasing the NO/NS ratio of AASC performance were worth investigating due to two main reasons. First, NaOH is a less expensive activator compared to Na_2_SiO_3_. Second, a high amount of Na_2_SiO_3_, which means a lower NO/NS ratio, leads to low concrete workability. This is an essential fresh property. Workability can affect casting, molding, and even all properties of concrete because concrete owes its durability to chemical reactions that start in the early hours when the binder reacts with alkaline solutions.

**Figure 9:**
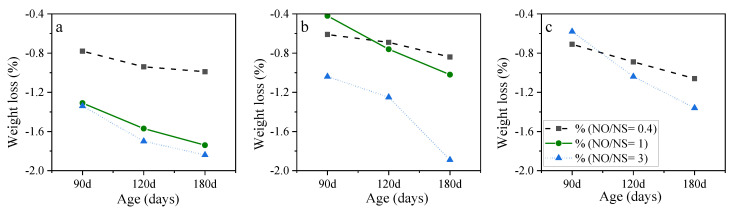
The effects of NO/NS ratios on weight loss at a molarity of (**a**) 6, (**b**) 10, and (**c**) 14.

Figure 8 and Figure 9 display the impacts of an increase in NO/NS ratio on the compressive strength reduction and weight loss reduction of AASC after acid exposure. At a molarity of 6, Figure 8a, the lowest NO/NS ratio (0.4) showed a 14.55% strength reduction after 180 days of exposure which was slightly lower than other modalities. As illustrated in Figure 8b,c, the higher NO/NS ratio resulted in higher compressive strength reduction for samples with molarities of 10 and 14. Regarding weight loss, the higher NO/NS ratio results in increasing weight loss of specimens as well. After 6 months of exposure, the weight loss values were 0.99%, 1.74%, and 1.84% for the ratios 0.4, 1, and 3. Therefore, the increase in the NO/NS ratio could reduce the production cost but worsen AASC performance in the acid attack. A high NO/NS ratio decreases Na_2_SiO_3_ content and increases NaOH weight in a concrete mixture, causing the reduction of silica, and an increase in free water in the mixture results in the creation of a weak AASC microstructure.

#### 3.2.4. Alkali Solution Content to Slag Ratio

**Figure 10:**
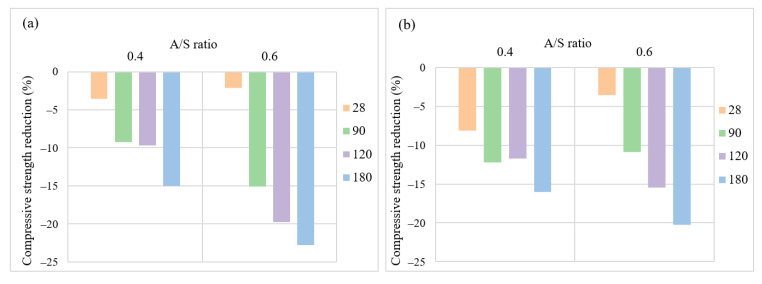
The effects of the alkaline solution to slag ratio on compressive strength (**a**) at a molarity of 6 and (**b**) at a molarity of 10 (A/S: the weight ratio of alkali solution content to slag).

**Figure 11:**
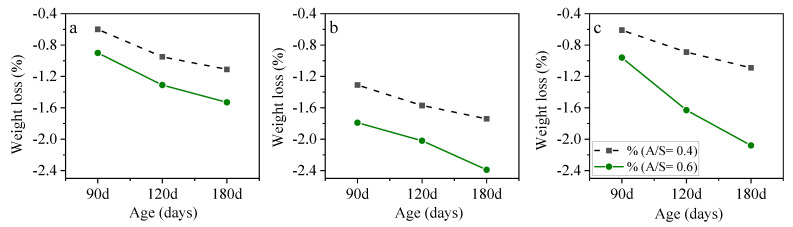
The effects alkaline solution to slag ratio on weight loss after acid exposure (**a**) molarity= 6 and NO/NS = 3, (**b**) molarity = 10 and NO/NS = 3, and (**c**) molarity = 10 and NO/NS = 1.

The effects of an increase in the A/S ratio from 0.4 to 0.6 on the compressive strength reduction at two molarities are shown in Figure 10. The higher A/S ratio means a higher alkaline solution in the mixture, and the results showed that the more alkaline solution leads to higher compressive strength reduction after 120 and 180 days of acid exposure. As shown in Figure 10a, at a molarity of 6 and A/S ratio of 0.6, AASC samples experienced roughly 15%, 20%, and 23% compressive strength reduction, respectively, after 90, 120, 180 days of exposure to acid, which is 5.8%, 10%, and 7.8% higher strength reduction compared to A/S ratio of 0.4 at the same ages. The same trend was observed at a molarity of 10 (Figure 7b) after 120, 180 days of exposure to acid. The results of weight loss are shown in Figure 11. This figure shows that after 180 days of exposure to acid, an increase in the A/S ratio increases weight loss from 1.11% to 1.53%. The same results were observed at a molarity of 10 and NO/NS ratio of 3, in which the weight loss increased from 1.09% to 2.08%. 

The main reasons for lower performance at a higher A/S ratio were insufficient binder volume in the mixture and excessive alkaline solutions consisting of free water. A higher A/S ratio led to a reduction of slag from 394 Kg/m^3^ to 345 Kg/m^3^ in one cubic meter of concrete, causing a reduction in the binder in the mixture.

### 3.3. Comparative Study on the Performance of AASC and OPC in Sulfuric Acid Attack

**Figure 12:**
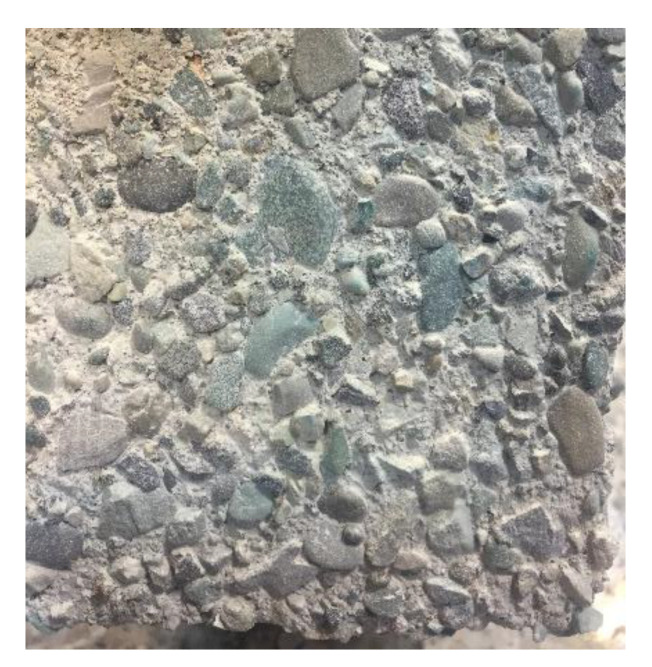
Visual inspection of OPC sample (100 × 100 mm) exposed to sulfuric acid after 6 months.

OPC samples were used as a reference to better understand AASC performance in the acid attack. As shown in Figure 12, the surface layers of OPC samples are completely removed, and aggregates are clearly visible.

**Figure 13:**
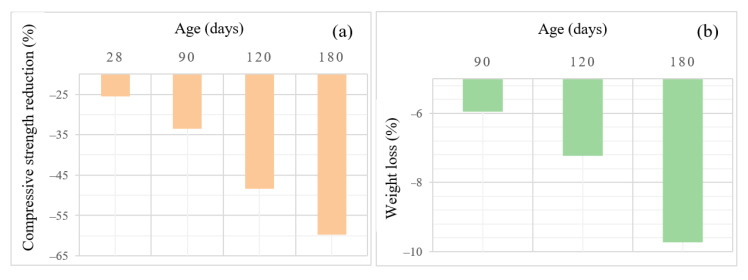
(**a**) Compressive strength reduction and (**b**) weight loss of OPC samples after exposure to acid for up to 6 months.

Figure 13 displays the strength reduction and weight loss of OPC samples immersed in the acid bath for up to 180 days. The average strength reduction for OPC samples was roughly 25.5% and 59.8% after 28 and 180 days of acid exposure, respectively. As explained in the previous section, for AASC samples, the lowest compressive strength reduction was 14.5% which is one-fourth of OPC concrete. The highest strength reduction for poorly designed concrete is 31.6% which is around half of OPC compressive reduction. The results revealed that cement-free concrete can achieve considerably lower strength reduction, especially at higher ages. In other words, selecting a proper mix of design parameters can sustainably impact the durability performance of concrete. Accordingly, a concrete element made by OPC concrete that deteriorated after two years due to acid attacks would acceptably work for at least 8 years if built by a well-designed AAS. Figure 13b shows the weight loss of OPC samples in sulfuric acid solution. The results showed that almost 10% of the weight was lost after 6 months of exposure to acid. For AASC, the lowest weight loss was 0.99%, roughly one-tenth of conventional concrete. The immersion of AASC in such a strong acid as H_2_SO_4_ caused decomposition of aluminum and destruction of Si-O-Al in AASC concrete leading to reducing mechanical properties as well as weight loss. 

The underlying reason for such a superior performance is attributed to the different microstructure of these two concretes. Cement is the most expensive and most vulnerable ingredient in conventional concrete. The acid attack on OPC includes attacks on hydration products and converting calcium-containing phases such as CH, CSH, and calcium aluminate hydrate to calcium salt. This reaction causes expansion, cracking, and deterioration of conventional concrete. The CaO content in cement and slag is 63.50% and 36.52% (Table 1). Hence, AASC contains considerably lower calcium-containing components compared to OPC. In addition, there is no sign of CH as a hydration product in the AASC structure. This phase that reacts with acid agents and forms calcium salts in conventional concrete does not play a role in the acid attack mechanism of AASC.

## 4. XRF Results

The XRF was conducted on the other layer, which was removed from the sample using sandpaper to determine its chemical composition. As discussed in the previous section, a soft white layer is formed on AASC samples after exposure to sulfuric acid. The XRF results are presented in Table 4. The results show that three main phases are SiO_3_, SiO_2_, and CaO, which consist of roughly 78% of the composition. It also should be mentioned that the powder did not dry before conducting the XRF test. Water content in the samples justifies high (14.86%) loss of ignition (LOI) values. The high percentages of SO_3_ are due to exposure of samples to sulfuric acid. The results reveal that the SiO_2_, Al_2_O_3_, and CaO contents are reduced compared to the chemical composition of slag, which indicates that acid agents break the silica-aluminate bonds, and these phases are leached out of the structure.

## 5. Conclusions

In this study, the impacts of four mixed design parameters on the performance of AASC exposed to sulfuric acid attack were investigated. For this purpose, samples were cured in water and immersed in acid baths and were tested on the same days to determine the compressive strength reduction as well as weight loss. Moreover, an XRF test was also conducted to understand the phase changes before and after acid exposure. The tests were conducted on specimens at 7 days for up to 180 days. Moreover, an OPC sample was utilized as a reference. The results reveal that the compressive strength reduction and weight loss of AASC samples compared to OPC concrete were near one-fourth and one-tenth, respectively. The main hydration products of AASC before and after acid exposure were the same, and there were no signs of gypsum and ettringite. While an increase in the weight ratio of NaOH to Na_2_SiO_3_ is economically desirable, it would not have any impact on either strength resistance or weight loss. 

The increase in the alkaline solution to slag ratio, which reduces the binder content, increased the compressive strength reduction and weight loss due to excessive alkaline solution in the mixture. This mixture has relatively high-water content. Furthermore, the application of KOH-activated slag concrete negatively affected mechanical performance and also increased the production cost of AASC. The reduction of SiO_2_, Al_2_O_3_, and CaO contents indicated the breaking of silica-aluminate bonds in structure and leaching of these components out of the concrete. AASC samples cured in water reached a high compressive strength at early ages and gained more than 70% of final compressive strength (compressive strength at 180 days) after 7 days. 

In future works, the authors recommend performing chloride permeability and water impermeability tests. The microstructure of AASC after acid attacks using thermogravimetric analysis and SEM is encouraged. The feasibility of the production of precast anti-acid AASC tiles should be investigated. Furthermore, the same studies should be done on one-part alkali-activated materials apart from the two-part alkali-activated materials investigated in this study.

## Figures and Tables

**Table 1:** Chemical compositions of OPC and GGBFS (wt.%).

Materials	CaO	SiO_2_	Al_2_O_3_	MgO	TiO_2_	MnO	S	K_2_O	Fe_2_O_3_	NaO_2_	SO_3_	LOI *
OPC	63.50	21.50	5.10	2.30	-	-	-	0.93	3.80	-	2.00	0.70
GGBFS	36.52	38.35	10.88	8.77	1.48	1.25	1.21	0.93	0.52	0.49	-	0.26

* Loss On Ignition.

**Table 2:** Aggregates physical properties.

Aggregate Type	Fineness Module	Sand Equality	Specific Gravity (SSD) (gr/cm^3^)	Water Absorption (%)
Fine	2.99	77	2.47	2.06
Course	-	-	2.59	0.76

**Table 3:** AASC mix designs (A/S: the weight ratio of alkaline solutions to slag, NO/NS and KO/NS: the weight ratio of NaOH (or KOH) to Na_2_SiO_3_).

Mix Code	Alkaline Solution	Molarity	A/S	NO/NS (KO/NS)	Alkaline Solution (Kg/m^3^)	Slag (Kg/m^3^)
N6041	NaOH	6	0.4	1	158	394
N6043	NaOH	6	0.4	3	158	394
N10041	NaOH	10	0.4	1	158	394
N10043	NaOH	10	0.4	3	158	394
N14041	NaOH	14	0.4	1	158	394
N14043	NaOH	14	0.4	3	158	394
N60404	NaOH	6	0.4	0.4	158	394
N100404	NaOH	10	0.4	0.4	158	394
N10061	NaOH	10	0.6	1	207	345
N6063	NaOH	6	0.6	3	207	345
N10063	NaOH	10	0.6	3	207	345
K6041	KOH	6	0.4	1	158	394
K6043	KOH	6	0.4	3	158	394
K10043	KOH	10	0.4	3	158	394
K10061	KOH	10	0.6	1	207	345

**Table 4:** Oxide composition of the surface layer of AASC samples after 180 days of exposure to acid (weight percentage).

CaO	SiO_2_	SO_3_	Al_2_O_3_	Fe_2_O_3_	MgO	K_2_O	NaO_2_	TiO_2_	P_2_O_5_	LOI
20.43	29.88	27.55	3.89	0.8	0.36	1.03	0.59	0.45	0.09	14.86

## Data Availability

Not applicable.
